# Notes and News

**Published:** 1896-01-04

**Authors:** 


### NOTES AND NEWS.

(COLLECTED AND COMMUNICATED.)

A NEW ward is to be opened early in the New Year at the Corbett Hospital, Brierley Hill. It will be used as a male medical ward, as it adjoins the accident ward, and thus enables the male patients to be accommodated on the same side of the building.

A BILL containing strict regulations relating to the sale of milk is being brought forward by the New York Board of Health. It is proposed to require every milk vendor to obtain a certificate from authorised examiners that their cattle are free from disease.

THE University of Pennsylvania has just been enriched by the addition of the Pepper Laboratory of Clinical Medicine, which was formally opened by Dr. Billings, director of the University Hospital, with which the laboratory is connected.

A NEW medical journal, devoted to the subject of children's diseases, is announced for January. The editor is Dr. George Carpenter, late resident medical officer at the Evelina Hospital for Children, and author of numerous works on the subject of the journal. The owner is Dr. Dillon Brown, of New York. The contributors to the first number are Drs. Jacobi, Phelps, Sutherland Manges, Frentveight, Lilienthal, and Collins.

WHAT should be a most useful institution, namely, a private hospital for women and children, has been opened in Berkeley Square, Bristol. Such institutions should offer opportunities for self-respecting persons who are able to pay to obtain the same advantages as the poor now have in hospitals, instead of usurping the place of the poorest who cannot pay the smallest sum. We think, however, that the title "Hospital" should be restricted to public institutions with a Committee of Management openly elected, and that if this is a private affair it should choose some other designation.

THE Queen has sent a gift of cast linen to the British Home for Incurables.

IT is proposed to establish a Bureau of Public Health for Pennsylvania, whose labours shall especially be directed towards checking the introduction and spread of epidemic diseases.

THE first list of signatures for the Mile End petition to the Charity Commissioners against the destruction of the Trinity Almshouses amounted to 1,100 names.

THE South-Eastern Railway Company have handed £221 to Guy's Hospital, the result of a concert given by the employés of the company in aid of the institution.

A STATUE has been erected of Dr. Müller, the first German professor at the Tokio Medical College. The statue, which is of bronze, has been executed by Mr. Fufita Bunzo, a Japanese sculptor of repute.

IT is stated that the governors of Guy's Hospital have sanctioned the proposals of the school staff to erect new school buildings attached to the medical school. The estimated cost of £12,000 is being privately subscribed.

THE twelfth International Congress of Medicine is to be held in August, 1897, at Moscow. The Grand Duke Sergi, Governor-General of Moscow, and uncle of the Czar, has consented to be patron, and it is understood the presidency will be accepted by Dr. Klein. The Russian Government has made a grant towards the expenses of the Congress. The official languages to be used are French and German.

THE trustees of the Bellahouston Fund have granted a further £2,500 to the Royal Glasgow Infirmary for improvements in the equipment of the medical school, and £7,500 for additions to the same; to the Western Infirmary £8,500 for structural additions; and to the Victoria Infirmary £6,000 for an out-patients' dispensary, to be called "The Bellahouston Dispensary." Mr. Cameron Corbett, M.P., has given 1,000 guineas towards the last object.

A MARBLE slab bearing an epitaph to Pasteur has recently been erected by the Municipal Council of Paris in the old laboratory of the Ecole Normale. The inscription runs as follows:—

"Ici fut le Laboratoire de Pasteur."
1837. Fermentations.
1860. Génération Spontanée.
1865. Maladies des Vins.
1868. Maladies des Vers à Soie.
1881. Virus at Vaccins.
1885. Prophylactie de la Rage.

IMPROVEMENTS and alterations are contemplated at the York County Hospital. One proposal embraces a scheme to remove the children's ward to the quarters at present inhabited by the nurses. At Huddersfield alterations and extensions have been effected at the infirmary in connection with the drainage, baths, washhouse, laundry, disinfection chamber, engine house, boiler house, and lavatories. At the Sussex County Hospital, Brighton, sanitary towers are to be erected, and the out-patients' department is to be removed from the present buildings.

